# Implantable vagus nerve stimulation system performance is not affected by internal or external defibrillation shocks

**DOI:** 10.1007/s10840-021-01050-7

**Published:** 2021-08-31

**Authors:** Imad Libbus, Scott R. Stubbs, Scott T. Mazar, Scott Mindrebo, Bruce H. KenKnight, Lorenzo A. DiCarlo

**Affiliations:** LivaNova USA, Inc., Houston, TX USA

**Keywords:** Autonomic regulation therapy, Vagus nerve, Vagus nerve stimulation, Heart failure, Implantable cardioverter defibrillator, Defibrillation, Autonomic nervous system

## Abstract

**Purpose:**

Autonomic regulation therapy (ART) for heart failure (HF) is delivered using vagus nerve stimulation (VNS), and has been associated with improvement in cardiac function and HF symptoms. VNS is delivered using an implantable pulse generator (IPG) and a lead placed around the cervical vagus nerve. Because HF patients may receive concomitant cardiac defibrillation therapy, testing was conducted to determine the effect of defibrillation (DF) on VNS system performance.

**Methods:**

Normal swine (*n* = 4) with VNS system implants on the right cervical vagus nerve received sequential defibrillation shocks with three defibrillation systems: an implantable cardioverter defibrillator (ICD), a subcutaneous ICD (S-ICD), and an external cardioverter defibrillator (ECD). Each system delivered a series of bipolar high-energy shocks and reverse-polarity high-energy shocks.

**Results:**

The specified cardiac defibrillation shocks were delivered successfully from each of the three defibrillation systems to all animals. After each shock series, interrogation of the IPG confirmed that software and data were unchanged from pre-programmed values. After all of the defibrillation shocks were delivered, the IPGs underwent and passed comprehensive electrical testing demonstrating proper system function. No shifts in IPG parameters or ART system failures were observed, and histologic evaluation of the vagus nerve revealed no anatomic changes.

**Conclusions:**

Implantable VNS systems were tested in vivo for immunity to defibrillation via ICD, S-ICD, and ECD, and were found to be unaffected by a series of high-energy defibrillation shocks. These results confirm that ART systems are capable of continuing to function after defibrillation and the cervical vagus nerve is anatomically unaffected.

## Introduction

Heart failure (HF) is characterized by hemodynamic abnormalities that are associated with a marked autonomic imbalance consisting of increased sympathetic activity and withdrawal of parasympathetic tone. This pathological adrenergic hyperactivation contributes to the progression of HF and increases the risk of mortality and morbidity independent of left ventricular ejection fraction (EF) and ventricular arrhythmias [1].

**Fig. 1:**
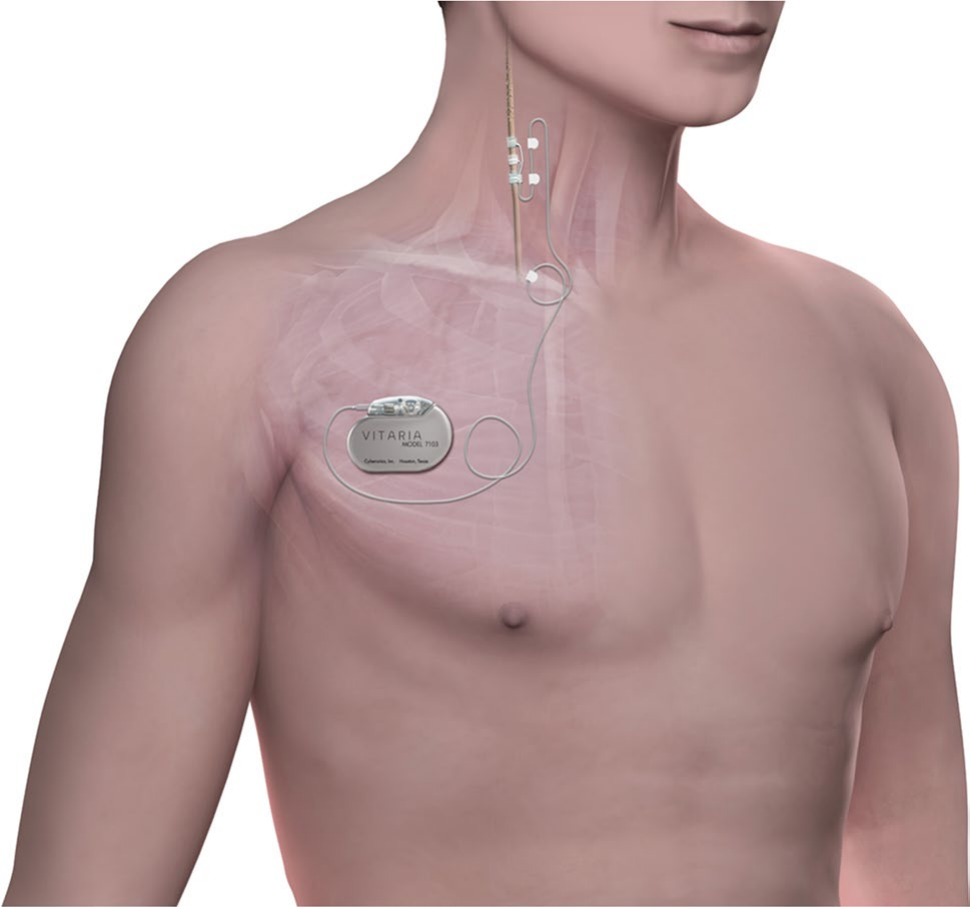
Autonomic regulation therapy (ART) system consisting of an implantable pulse generator and a lead that is placed around the right cervical vagus nerve

Autonomic regulation therapy (ART) is a novel therapy for the management of HF. ART uses cervical vagus nerve stimulation (VNS) to increase parasympathetic activity and to restore autonomic balance. ART is delivered using chronic stimulation through an electrical lead that is implanted around the cervical vagus nerve without requiring any mapping for placement [2]. An ART system consists of a self-sizing, atraumatic helical lead that is placed around the cervical vagus nerve without requiring any mapping for placement and has a low rate of complications and failures [3]. The lead is connected to an implantable pulse generator (IPG) that is implanted in an infraclavicular subcutaneous pocket and delivers neurostimulation according to programmed stimulation parameters (current amplitude, pulse width, frequency, and duty cycle) that can be adjusted wirelessly by inductive telemetry using a handheld wand and programming computer (Fig. 1).

In the ANTHEM-HF study, ART delivered using open-loop VNS was associated with long-term improvement in left ventricular function, 6-min walk distance, NYHA class, heart rate, heart rate variability, and quality of life in patients with HF and reduced EF (HFrEF) [4–6]. The impact of ART on mortality and morbidity is being evaluated in an ongoing pivotal study in patients with HFrEF [7].

International guidelines for the treatment of HF recommend implantable cardioverter defibrillator (ICD) therapy for patients with HFrEF and at risk for ventricular arrhythmias [8–11]. Because HFrEF patients may be recipients of both ART and ICD and exposed to either external or internal high-energy defibrillation, it is important to establish whether defibrillation shocks at industry-maximum levels may have an effect on the operation of ART systems. ART systems are designed with high voltage protection, and previous benchtop testing according to the international standard for determining defibrillation immunity of implantable medical devices (ISO 14,708–1) [12] established that implantable VNS systems were unaffected by high-energy defibrillation in vitro [13].

The purpose of this study was to determine whether functional performance of ART systems is detrimentally affected by internal and external defibrillation in vivo using defibrillation shocks delivered by an ICD, a subcutaneous ICD (S-ICD), and an external cardioverter defibrillator (ECD).

## Methods

The study was approved by the American Preclinical Services Institutional Animal Care and Use Committee (IACUC) and conformed to the National Institutes of Health *Guide for the Care and Use of Laboratory Animals*. Normal Yorkshire swine (*n* = 4) weighing 40 to 50 kg were implanted with the VITARIA® ART system (LivaNova USA, Houston, TX; Fig. 1), consisting of a Model 7304 lead that was placed around the right cervical vagus nerve and a Model 7103 implantable pulse generator (IPG) that was placed in a subcutaneous pocket in the animal’s right dorsolateral neck area, as previously described [14]. All procedures were performed under general anesthesia and sterile conditions.

After implantation, the IPG was activated and programmed to deliver chronic vagus nerve stimulation using 2.5 mA amplitude, 250 µsec pulse width, 5 Hz frequency, and a duty cycle of 14 s on/66 s off; these parameter values are typical in clinical setting.

**Fig. 2:**
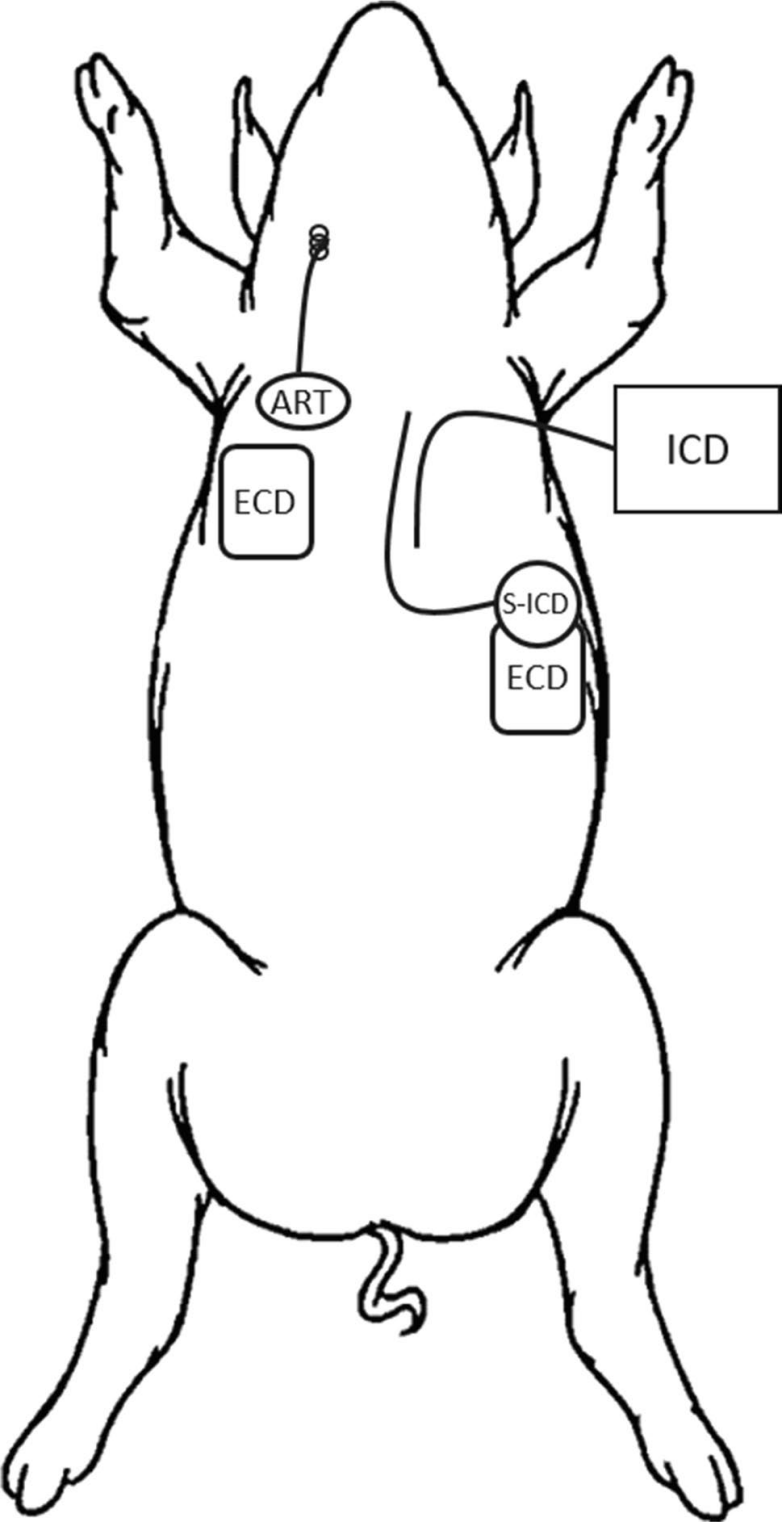
Placement of the ART system and the three defibrillation systems. Each of the defibrillation systems was sequentially implanted, used, and then explanted prior to the implantation of the next system

For each of the four animals, the following three defibrillation systems were sequentially implanted, used, and then explanted prior to the implantation of the next system. The placement of the implants is shown in Fig. 2.

1. ICD (Spring Quattro Secure S Ventricular Lead DF4, Medtronic, Minneapolis, MN). The ICD lead was implanted transvenously into the right ventricular apex under fluoroscopic guidance and connected to an external pulse generator, which delivered defibrillation shocks at 50 J.
2. S-ICD (Emblem S-ICD, Boston Scientific, Saint Paul, MN). The lead was implanted subcutaneously in the standard L-shaped configuration [15]. A stainless steel disk the size of the S-ICD IPG (70 mm diameter, 13 mm thick) was implanted in the left thoracic region and used as a return electrode. Primary (coil A) and secondary (coil B) defibrillation vectors were sequentially tested. The electrodes were connected to an external pulse generator, which delivered defibrillation shocks at 75 J.
3. ECD (M-Series Biphasic Defibrillator, Zoll Medical, Boston, MA). Defibrillation pads (40/50 cm^2^) were placed on the chest in the conventional anterior configuration (right pectoral to left thoracic), and defibrillation shocks were delivered at 200 J.

The order in which the three defibrillation systems (ICD, S-ICD, and ECD) were used was randomly selected for each animal using a six-sided die to select among the six possible options. In each configuration, a series of high voltage biphasic defibrillation pulses were delivered: three pulses separated by 20 s, followed by a 60-s pause, followed by three pulses separated by 20 s with reverse polarity. The application of the defibrillation pulses was not synchronized to the output of the VITARIA IPG.

Each IPG was interrogated and the programmed device parameters were compared before and after each set of shocks. After all of the planned shock applications were delivered, each IPG underwent evaluation for damage using electrical testing that duplicated what is performed at the end of the manufacturing process for ART systems to confirm that each ART system met all of the required functional specifications, as previously described [16].

To confirm that autonomic function was intact, acute heart rate dynamics were assessed in response to vagus nerve stimulation, as previously described [17]. VNS intensity was sequentially raised from 0 to 3.0 mA in 1 mA increments, and acute heart rate change derived from the ECG was measured. This assessment was performed before and after the series of defibrillation shocks.

At the end of the procedure, the right and left cervical vagus nerves were removed for histology assessment. The entire nerves were harvested from the thoracic inlet to the upper neck, immersion fixed in 10% neutral buffered formalin, and embedded in paraffin. Right cervical nerve sections were taken at sites proximal to the electrode, three sections underneath the electrodes, and distal to the electrode. Nerves sections were stained with hematoxylin and eosin (H&E) and Luxol fast blue (LFB) and evaluated by an independent study pathologist.

## Results

The order in which the three defibrillation systems were randomized in each animal is shown in Table 1. All defibrillation tests were performed successfully in all animals, with no deviations from the protocol.

**Table 1:** Randomized order of defibrillation application

Animal number	1st defibrillation	2nd defibrillation	3rd defibrillation
1	S-ICD	ECD	ICD
2	ICD	ECD	S-ICD
3	S-ICD	ICD	ECD
4	ECD	ICD	S-ICD

In two of the animals, a defibrillation shock induced ventricular fibrillation, and an additional external defibrillation shock was required to terminate the arrhythmia. In total, each animal implanted with the VITARIA system received a total of 24–26 high-energy shocks through either an ICD, S-ICD, or ECD configuration.

After each set of shocks, each IPG was interrogated, and the interrogated data (serial number, patient ID, output current, frequency, pulse width, on-time, and off-time) was found to be unchanged from the programmed values prior to the shocks. Comprehensive electrical testing of each ART system after delivery of the final shock confirmed no device failures or parameter shifts and that they were still within the manufacturer’s functional specifications. The output of the pulse generator was measured and found to be within the manufacturing tolerance for each programmed stimulation parameter.

An intensity-dependent heart rate response was observed before and after the series of defibrillation pulses, indicating the presence of autonomic engagement. The application of repeated shocks altered the autonomic conditions, resulting in an attenuated response post-defibrillation (from a peak response of 5.5 ± 3.1 bpm to a peak response of 2.1 ± 6.4 bpm).

**Fig. 3:**
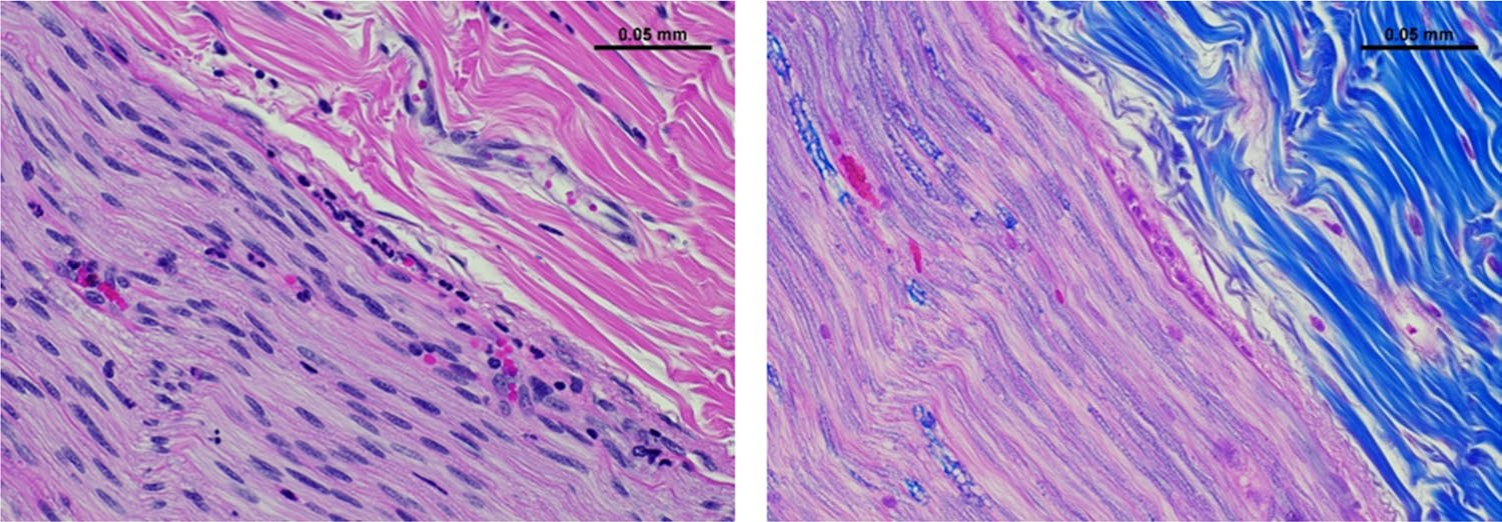
Hematoxylin and eosin (H&E, left panel) and Luxol fast blue (LFB, right panel) staining of longitudinal sections of the right vagus nerve at the electrode site from a representative animal, showing scattered, minimal to mild amounts of neutrophils in the epineurium, perineurium, and nerve

Histological analysis with H&E and LFB of the left and right cervical vagus nerves confirmed the absence of any damage to the nerve (Fig. 3). The nerves from all four animals showed no necrosis and minimal inflammation and hemorrhage. There was no minimal evidence of thermal injury or collagen degradation. There was no difference between histopathologic parameters in samples taken from the left and right nerve.

## Discussion

ART is a novel HF therapy that was shown in the ANTHEM-HF study to significantly improve cardiac function and reduce heart failure symptoms in patients with HF and reduced EF [4, 5, 18]. Preclinical studies suggest that the beneficial effects of ART derive from multiple synergistic mechanisms in which chronic ART improves regulatory control of the autonomic nervous system. VNS inhibits neural release of norepinephrine at cardiac effectors [19], restores autonomic balance as reflected in improvements in heart rate variability and baroreflex sensitivity [20, 21], reduces systemic inflammation [22–24], increases coronary flow [25], is anti-apoptotic [20, 26], directly modulates reflex processing within peripheral ganglia of the cardiac nervous system [27], and has anti-arrhythmic effects [21, 28–30, 32, 32, 33].

Because patients with HF and reduced EF are at risk for life-threatening ventricular arrhythmias, these patients commonly receive implantable defibrillator therapy (either ICD or S-ICD) and external defibrillation. As these patients may also receive neurostimulation for HF using an implantable system, it has been important to determine that such systems are able to survive the application of DF energy and continue to perform as originally programmed. Previous benchtop testing according to international testing standards established that implantable ART systems are unaffected by high-energy defibrillation in vitro [13].

In this in vivo study, the VITARIA ART system was evaluated after repeated defibrillation shocks were applied to electrodes in and on the thorax using ICD, S-ICD, and ECD systems. In each configuration, three sequential shocks were delivered, followed by three sequential reverse-polarity shocks. System programming and comprehensive electrical testing confirmed that the implanted ART system was unaffected by the applied energy, and continued to function normally throughout and after the procedure. Analysis of autonomic engagement and nerve histology showed that the vagus nerve was not damaged by defibrillation.

In addition to evaluating the effect that defibrillation has on the ART system, it is also important to evaluate the effect that ART stimulation has on cardiac sensing of ICDs, cardiac pacemakers, and cardiac resynchronization therapy (CRT) devices. This testing was previously performed and published [34], and demonstrated that maximum ART stimulation intensity did not result in a detectable level of interference with either bipolar and unipolar sensing.

## Conclusions

An implantable ART system was tested in vivo for immunity to defibrillation via ICD, S-ICD, and ECD, and was found to be unaffected by a series of high-energy defibrillation shocks. The study results confirm that ART systems are capable of continuing to function appropriately after defibrillation and the cervical vagus nerve is unaffected.
